# Systematic analysis of outcome indicators in traditional Chinese medicine clinical research for dry eye: A review

**DOI:** 10.1097/MD.0000000000043988

**Published:** 2025-08-29

**Authors:** Kang Tong, QiaoYing Lian, Wen Tang, Xingyu Chen, Fang Chen, Zhibin Wang, Shijie Qiao, Hairui Han, Chaoyang Yang

**Affiliations:** a College of Traditional Chinese Medicine, Fujian University of Traditional Chinese Medicine, Fuzhou, China; b Department of Traditional Chinese Medicine, Xiamen Third Hospital, Xiamen, China.

**Keywords:** clinical research, core outcome set, dry eye, methodology, outcome indicators, traditional Chinese medicine

## Abstract

This narrative review systematically analyzes outcome indicators in clinical research on traditional Chinese medicine (TCM) interventions for dry eye, aiming to analyze current methodological patterns and establish foundations for core outcome set development. Through comprehensive searches across Chinese (China National Knowledge Infrastructure, VIP Database for Chinese Technical Periodicals, Wanfang) and English databases (PubMed, Cochrane Library, Embase), 863 clinical studies were identified and analyzed for outcome indicator usage. Forty-six distinct outcome indicators were documented, cumulatively appearing 3885 times across 6 categories. The most frequently employed metrics were tear film break-up time (772 times), Schirmer test (753 times), and corneal fluorescein staining (559 times). Significant methodological concerns emerged, including marginalization of TCM-specific indicators, nonstandardized outcome reporting, substantial heterogeneity in indicator combinations lacking theoretical justification, poor clinical applicability, and absence of economic evaluations. The findings underscore the urgent need to develop a standardized core outcome set for TCM dry eye research to enhance methodological rigor, strengthen evidence synthesis, and optimize the integration of TCM’s therapeutic advantages in dry eye management.

## 1. Introduction

### 1.1. Rationale

Dry eye is a common multifactorial ocular surface disease in ophthalmology at present. Its own dryness, foreign body sensation, burning sensation, photophobia, blurred vision, asthenopia and other ocular surface discomfort symptoms seriously affect the quality of daily life.^[1]^ According to the 2021 dry eye Global epidemiological survey, the prevalence of dry eye in the world is 11.59%. Among them, the prevalence in East Asia is 42.8%, and the prevalence in China shows an increasing trend.^[2]^ However, the main treatment methods adopted by modern medicine are lubrication of the ocular surface, promoting repair, promoting tear secretion, anti-inflammatory and antibacterial treatment.^[3]^ The above treatments cannot fundamentally improve the symptoms of dry eye in patients, and long-term use of drugs leads to the occurrence of toxic side effects and is prone to recurrent symptoms.^[4]^

Dry eye belongs to the category of “white astringency” in traditional Chinese medicine (TCM).^[5]^ TCM believes that external dryness, aging, liver depression and other causes, resulting in internal injury of body fluid and blood, eye loss and nourishment, leading to the occurrence of dry eye.^[6]^ Traditional Chinese medicine has a good effect in the treatment of dry eye. The Guidelines for Clinical Application of TCM in the Treatment of dry Eye^[7]^ recommend Qiju Dihuang pill, Htuynia cordata eye drops, and compound wild chrysanthus eye plaster as the recommended Chinese patent medicine for dry eye, which can prolong the tear film breakup time, improve the stability of the tear film, and relieve the symptoms of dry eye.^[8]^ In addition, a variety of TCM treatment methods such as decoction, acupuncture, massage, drug fumigation and hot compress can be used.^[9]^ Therefore, TCM has obvious advantages over modern medicine in the treatment of dry eye. However, the problem is that at present, the outcome indicators of TCM treatment of dry eye are mainly based on western medicine indicators such as Schirmer secretion test and tear film breakup time,^[10–12]^ lacking core outcome indicators with TCM characteristics, which cannot truly reflect the advantages of TCM therapy in the treatment of dominant diseases of dry eye.

Core outcome sets is a unified and standardized minimum index set that must be measured and reported in clinical research of specific diseases at present,^[13]^ which is convenient for the comparison and combined analysis of dry eye TCM clinical research results, optimizing index selection and reducing the use of inappropriate evaluation indicators, and providing standard and standardized measurement indicators for dry eye TCM clinical research.^[14]^

### 1.2. Objectives

In this study, COS-STAR,^[15]^ The COMET Handbook: version 1.0^[16]^ and COS-STAD^[17]^ and other core index set guidelines developed research programs. Based on clinical research literature on dry eye treated by TCM therapy, the current application status of outcome indicators in dry eye treated by TCM therapy was summarized, so as to provide index sources for the next construction of dry eye TCM core index set.^[13]^

## 2. Methods

### 2.1. Inclusion criteria

The inclusion criteria were designed according to the principle of “PICOS” combined with the Reporting Standards for Clinical Randomized Controlled Trials of TCM,^[18]^ including population, intervention, comparison and study design.

(1) Population: dry eye patients who met the diagnostic criteria of the 2020 Chinese Expert Consensus on dry eye.^[19]^
(2) Intervention: including all TCM for dry eye treatment, such as Chinese medicine, acupuncture, massage, scraping and so on.
(3) Comparison: no restrictions.
(4) Study design: The clinical research articles on TCM therapy for dry eye published in current Chinese and English databases were selected, that is, the articles containing clear TCM therapy name and dry eye disease name and type were selected.

### 2.2. Exclusion criteria

The research type was basic research on the mechanism of drugs or acupuncture, including basic literature such as animal experiments, cell experiments or molecular biology experiments. Meta literature or review literature of TCM therapy for dry eye.

### 2.3. Information sources

The retrieval database covered China National Knowledge Infrastructure, VIP Database for Chinese Technical Periodicals database, Wanfang Data Knowledge Service Platform Chinese database, PubMed, Cochrane library, and Embase English database, and the retrieval time was from the establishment of each database to August 2023.

### 2.4. Search strategy

(1) The Chinese search keywords were determined as “dry eye,” “dry eye disease,” “white astrinusitis,” “TCM,” “TCM” and “clinical research” by means of subject words combined with free words. The English search keywords were “Dry eye,” “Dry Eye Disease,” “Chinese Medicine,” “Chinese Medicine,” “Clinical Studies.”
(2) Chinese search format: (“ dry eye “or” dry eyes “or” white dry syndrome “) merger (“ Chinese medicine ‘or’ Chinese medicine ‘or’ Chinese medicine ‘or’ formula ‘or’ native ‘or’ acupuncture ‘or’ needle ‘or’ moxibustion ‘or’ (massage) ‘concentration’ clinical trials ‘or’ clinical research ‘or’ clinical observation ‘or’ effect ‘or’ curative effect evaluation ‘or’ curative effect analysis ‘or’ a randomized controlled trial” “Or” effect observation “)
(3) English search format: (“ Dry eye “or” Dry eyes “or” White dry syndrome “) merger (the “Chinese medicine” or “Chinese medicine” or “Chinese medicine” or “formula” or “native” or “acupuncture” or “needle” or “moxibustion” or “massage”) merger (“ clinical trials ‘or’ clinical research ‘or’ clinical observation ‘or’ effect ‘or’ curative effect evaluation ‘or’ curative effect analysis ‘or’ a randomized controlled trial” Or “Effect observation”)

### 2.5. Selection process

The data extraction table was designed according to the principle of “PICOS” combined with the Reporting Standards for Clinical Randomized Controlled Trials of TCM.^[17]^ Two researchers independently screened the literature according to the inclusion and exclusion criteria of the literature, and the double entry method was adopted for screening. At the same time, the data were extracted into the data extraction table.

### 2.6. Data collection process

Two researchers extracted 7 aspects including literature publication time, sample size, research topic, research object, intervention measures, control measures and evaluation indicators. If there was any disagreement, the third researcher would solve the problem through consultation.

### 2.7. Data processing and statistical analysis

The outcome indicators in the extracted data were standardized according to the national textbook of TCM Ophthalmology,^[6]^ the International Clinical Practice Guideline of TCM Dry Eye (2021-12-14)^[5]^ and the 2020 Chinese Expert Consensus on dry eye.^[19]^ The outcome indicators were divided into 6 index domains, including symptoms and signs, meibomian gland function, TCM indicators, quality of life, physical and chemical indicators, and safety indicators, according to the “Technical Specification for the Development of core outcome sets of Clinical Trials of TCM.”^[20]^

## 3. Results

### 3.1. Study selection

**Figure 1.**
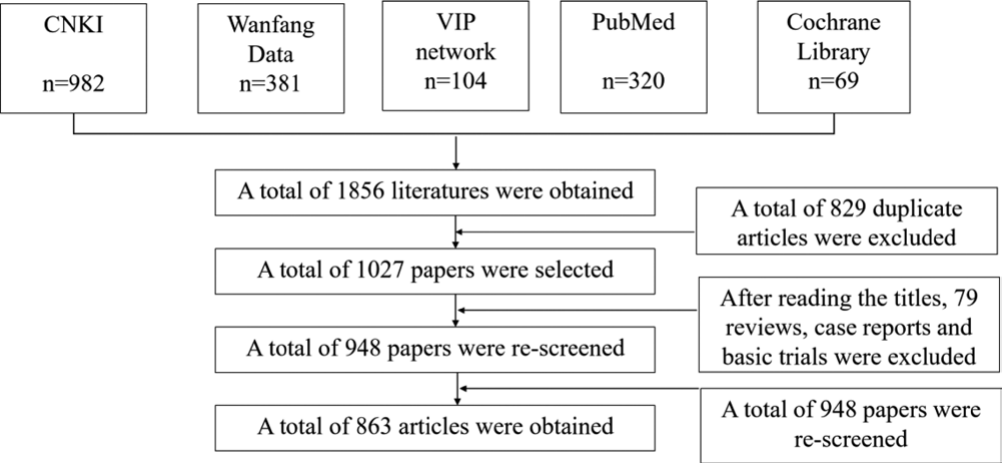
Literature screening process. CNKI = China National Knowledge Infrastructure, VIP = VIP Database for Chinese Technical Periodicals.

A total of 1856 Chinese and English databases were retrieved in this study, 829 duplicate literatures were eliminated through rechecking, and 1027 literatures were obtained for initial screening. After reading the title, 79 reviews, case reports and basic experiments were excluded, 948 literatures were obtained for secondary screening, and 948 literatures were carefully read for full text, and 863 literatures were obtained. See Figure 1 for details of the screening process.

### 3.2. Study characteristics

A total of 863 Chinese medicine clinical trials of dry eye were included, with a total of 77,751 patients. The sample size ranged from 8 to 767, and the average sample size of each clinical study was 90.

#### 3.2.1. *Literature year*

**Figure 2.**
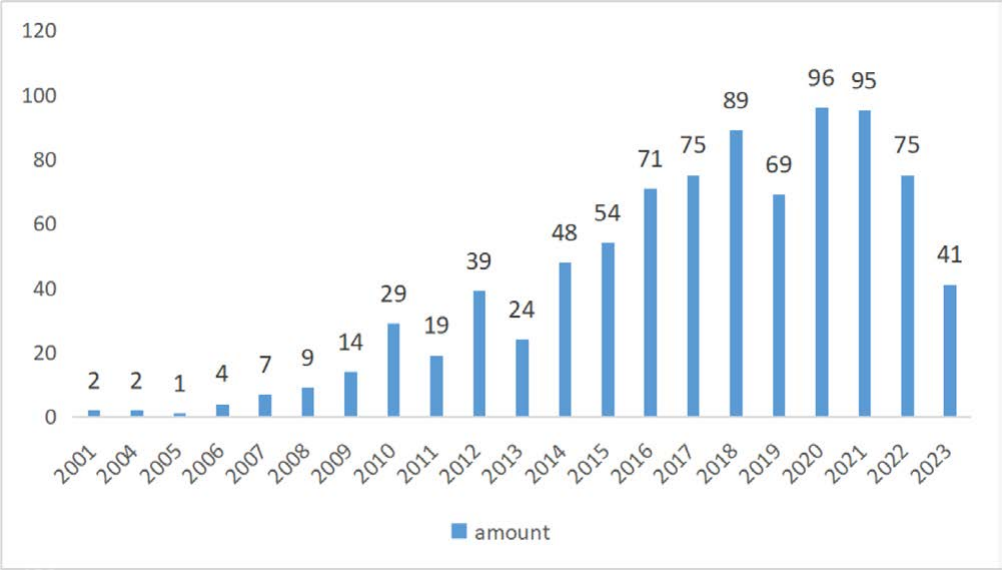
Distribution of published years of TCM clinical studies on dry eye. TCM = traditional Chinese medicine.

According to the inclusion criteria and the above screening process, a total of 863 clinical research articles were finally included. The articles were published from 2001, with the lowest number in 2005 and the highest number in 2020, with 96 articles published, an average of 41 articles per year. From 2001 to 2020, it showed an upward trend, and from 2020 to 2023, it showed a downward trend. Figure 2 shows the distribution of literature publication years.

## 4. Results of syntheses

### 4.1. *Outcome indicators*

A total of 46 outcome indicators (3885 in total) were used in 863 studies. The minimum number of indicators in a single clinical study was 1, and the maximum number was 10, with an average of 4.5 indicators per study. The top 10 most frequently used indicators were: Tear film breakup time (772 times), Schirmer secretion test (753 times), Corneal fluorescein staining (559 times), effective rate (542 times), clinical symptom score (304 times), clinical symptom score (137 times), OSDI scale (134 times), TCM syndrome score (121 times), tear meniscus height (61 times), interleukin (55 times), each indicator was classified, and the classification of specific outcome indicators is shown in Table 1.

**Table 1:** Classification of outcome indicators.

Classification	Name of index	Frequency	Classification	Name of index	Frequency
Symptoms and signs	Effective rate	542	–	The Hamilton depression scale	2
	Clinical symptom score	304	–	Visual-related quality of survival grade	1
	Clinical symptom	137	–	self-rating depressive scale	1
	Visual analogue scale grade	21	–	The Pittsburgh sleep quality scale	1
	Vision acuity	20	Physicochemical indexes	Tear film breakup time	772
	Intraocular pressure	5	–	Schirmer test	753
Glandulae tarsals function	Meibomian gland secretion trait score	48	–	Corneal fluorescein staining	559
	Meibomian gland secretion ability score	24	–	Tear meniscus height	61
	Meibomian gland deletion score	17	–	interleukin	55
	Meibomian gland opening blockage score	14	–	Tumor necrosis factor-α	40
	Eyelid margin morphology score	14	–	Classification of conjunctival congestion	17
	The score of eyelid fat discharge	6	–	Corneal surface regularity index	12
Traditional Chinese medicine index	Traditional Chinese medicine syndrome score	121	–	Corneal surface irregularities index	11
	TCM symptoms and signs points	37	–	Matrix metalloproteinase-9	9
	OSDI scale	134	–	Epidermal growth factor	3
Quality of life	25-item national eye institute visual function questionnaire	27	–	Tear lactoferrin	2
	SPEED questionnaire	10	–	Lissamine green staining	1
	the MOS item short form health survey (SF-36)	9	–	Rose Bengal staining	1
	Chinese dry eye questionnaire	6	Safety index	Adverse effects	46
	Quality of life for patients with visual impairment	4	–	Incidence of adverse reactions	29
	Self-rating anxiety scale	3	–	Safety evaluation grade	1
	Quality of life core scale	2	–	adverse event	1
	Hamilton anxiety scale	2	–	–	–

### 4.2. Quantity of indicators used

A total of 863 studies were included, and 41 studies used 1 outcome index, of which 40 were effective rate and 1 was schirmer test. 821 studies used 2 to 10 indicators at the same time, of which 276 studies used 5 indicators at the same time, accounting for the highest proportion, and 1 study used 1 indicator, accounting for the lowest proportion. See Table 2 for the distribution of the combined use of specific indicators.

**Table 2:** Distribution of the number of indicators used.

Number of indicators used	Frequency	Proportion	Number of indicators used	Frequency	Proportion
1	41	4.8%	6	98	11.4%
2	27	3.1%	7	44	5.1%
3	105	12.2%	8	17	2.0%
4	246	28.5%	9	8	0.9%
5	276	32.0%	10	1	0.1%

### 4.3. Analysis of index association rules

(1) The indicators of 863 clinical research literatures were applied to the Apriori algorithm of SPSS Modeler 18.0 software, and the support ≥20%, confidence ≥80%, maximum number of preceding items was 2, and promotion >1 were set. The association rules of all indicators were analyzed, and a total of 19 association data were obtained, including 7 2-type association rules. Among them, the combination of indicators with the highest support was Schirmer test and breakup time. The support showed that at least 89% of the literatures used both indicators, and the probability of using Schirmer test and breakup time was at least 93%. See Table 3 for details.
(2) There were 11 triple association rules, of which the indicator combination with the highest support was Schirmer test and tear film breakup time. The support showed that at least 63% of the literatures used the 3 indicators at the same time, and the probability of using Schirmer test and Corneal fluorescein staining at the same time was at least 97%. See Table 4 for details.

**Table 3:** Dry eye literature indicators double rule situation.

Serial number	Consequent	Antecedent	Support %	Confidence %
1	Schirmer test	Tear film breakup time	89.49	93.56
2	Schirmer test	Corneal fluorescein staining	65.20	96.71
3	Tear film breakup time	Corneal fluorescein staining	65.20	97.23
4	Schirmer test	effective percentage	61.24	85.42
5	Tear film breakup time	effective percentage	61.24	86.16
6	Schirmer test	Clinical symptom score	34.35	92.76
7	Tear film breakup time	Clinical symptom score	34.35	93.42

**Table 4:** Triad rules of dry eye literature indicators.

Serial Number	Consequent	Antecedent	Support %	Confidence %
1	Tear film breakup time	Schirmer test + Corneal fluorescein staining	63.05	97.49
2	Schirmer test	Efficiency rate + Tear film breakup time	52.77	94.86
3	Tear film breakup time	Effective rate + Schirmer test	52.32	95.68
4	Schirmer test	Effective rate + Corneal fluorescein staining	37.40	98.19
5	Tear film breakup time	Effective rate + Corneal fluorescein staining	37.40	96.37
6	Schirmer test	Clinical symptom score + Tear film breakup time	32.09	95.07
7	Tear film breakup time	Clinical symptom score + tear secretion test	31.86	95.74
8	Schirmer test	Clinical symptom score + Corneal fluorescein staining	25.76	96.93
9	Tear film breakup time	Clinical symptom score + Corneal fluorescein staining	25.76	96.93
10	Schirmer test	Clinical symptom score + Efficiency rate	20.23	93.30
11	Tear film breakup time	Clinical symptom score + Efficiency rate	20.23	94.41

## 5. Discussion

A total of 6 Chinese and English databases were searched, and the inclusion and exclusion criteria were set for the included clinical studies of dry eye TCM. A total of 863 clinical studies were collected, and the outcome indicators were analyzed, and it was found that there were some problems such as serious marginalization of TCM indicators, nonstandard expression of outcome indicators, large heterogeneity of outcome indicators combination and lack of logical support, poor clinical practicality of indicators and lack of economic indicators.

### 5.1. TCM indicators are seriously marginalized

In terms of the types of indicators used, as western medicine physical and chemical indicators, tear film breakup time was used 772 times, Schirmer test was used 753 times, and corneal fluorescein staining was used 559 times. At the same time, in the correlation analysis of indicators, the 2-rule combination of tear film breakup time and Schirmer test was used most frequently. The triple rule combination of tear film breakup time, Schiram secretion test and corneal fluorescein staining has the highest utilization rate, which is in line with the objective diagnostic indicators of the current diagnostic criteria of “Chinese Expert Consensus on dry eye.”^[19]^ However, TCM indicators such as TCM syndrome score or TCM symptom score were used 152 times, while clinical symptom score and clinical symptoms were used 304 times and 137 times respectively as clinical symptom response indicators of dry eye patients, and the frequency was higher than that of TCM indicators. The subjective indexes of macroscopic symptoms and signs and the microscopic physical and chemical indexes all rely on the evaluation indexes of modern medical ophthalmology. The TCM syndrome indexes that truly reflect the curative effect of TCM are rarely used, resulting in the inability to truly reflect the efficacy of TCM interventions in the diagnosis and treatment of dry eye.

### 5.2. Nonstandard expression of index terms

There are a large number of nonstandard expressions in the outcome indicators. Among them, the “Schirmer test” indicators in the category of physical and chemical indicators include “Schirmer test,” “Schirmer examination,” and so on. The “noncontact tear breakup time” in “tear breakup time” includes “noncontact tear breakup time” and “noninvasive tear breakup time.” In the category of symptoms and signs, the index of “clinical symptoms” has various expressions such as “subjective symptoms,” “dry eye symptoms,” “ocular symptoms,” “ocular local symptoms,” and “ocular subjective symptoms.” There are various expressions of “clinical symptom score” such as “subjective symptom score,” “ocular symptom score,” “symptom score,” “dry eye clinical symptom score” with the same meaning but different appellations. Therefore, it is necessary to further standardize the terminology of outcome indicators in secondary studies of clinical research. It also increases the difficulty in the classification of outcome indicators.

### 5.3. Large heterogeneity of indicator combination and lack of logical support

The 863 studies involved 46 kinds of indicators, and the number of indicators used ranged from 1 to 10. Among them, 41 studies used at least 1 indicator, of which 40 studies were effective indicators, and 1 study used 10 indicators, indicating that the current clinical research of dry eye TCM has great heterogeneity in indicator selection. At the same time, according to the association rule analysis, at least 89% of the literatures used Schirmer test and breakup time, and at least 63% of the literatures used 3 evaluation indicators, Schirmer test and corneal fluorescein staining, but in fact only 2 literatures used Schirmer test and breakup time. Fourteen articles only used 3 evaluation indicators, Schirmer test, tear film breakup time and corneal fluorescein staining, indicating that the combination logic of outcome indicators was highly random and lacked scientific and standardized index combination logic, which directly led to the difficulty of combining similar clinical studies for clinical research.

### 5.4. Poor clinical practicability of indicators

Some studies used matrix metalloproteinase-9, epidermal growth factor, tear lactoferrin and other tear component analysis as indicators. This study was mainly used for the detection of tear osmolality, but from the perspective of ophthalmic clinical practicability, it is necessary to use the test paper placed under the conjunctival sac or the lower eyelid capillary to absorb tears. In the process of tear collection, the patient’s comfort is poor, and the patient’s high cooperation is required to effectively collect tears. Therefore, the relevant examinations are rarely carried out in clinical practice. To some extent, the outcome indicators selected in the current clinical research of dry eye TCM lack clinical practical evaluation, and it is also a waste of scientific research resources.

### 5.5. Lack of economic indicators

At present, relevant foreign studies show that from 2015 to 2016, the national expenditure on ophthalmic drugs in the United States increased from US $3.39 billion to US $6.08 billion, of which dry eye accounted for 29.5%,^[20]^ so dry eye patients brought a huge burden to the country in terms of drug treatment. Compared with the normal population, patients with dry eye are restricted and affected in eye work due to their own discomfort such as dry eye, foreign body sensation and burning sensation, which brings great economic pressure to individuals and families.^[21]^ However, the included studies did not report economic indicators, indicating that economic factors have not been paid attention to in the clinical research of dry eye at present, and the TCM therapies, including Chinese medicine, acupuncture, moxibustion, acupoint iontophoresis, auricular point sticking, periocular point massage, scraping and fumigation, have unique advantages in the treatment of dry eye. It is necessary to compare the economic differences between Chinese and western medical therapies in the treatment of dry eye. Therefore, it is necessary to include economic indicators for measurement.

### 5.6. Limitations of the study

In the clinical research literature of dry eye, this study set up relatively strict inclusion and exclusion criteria, and the inclusion time was from the establishment of the database to August 2023. Clinical research protocols in clinical trial registration platforms in China and the United States were not retrieved in this study, and the outcome indicators may be missing. In terms of index analysis, no horizontal comparative analysis was used for indicators of different dry eye types or different treatments, which could not reflect the degree of selection change of outcome indicators at different measurement nodes. Therefore, the analysis results of this study have limitations in the inclusion and selection of outcome indicators. In the next step, the above defects will be more strictly standardized. Through doctor-patient interviews, Delphi method and consensus meeting, the core outcome sets of dry eye TCM will be constructed, and the efficacy evaluation method of dry eye TCM clinical research will be formed.

## 6. Conclusion

This study analyzed the clinical research of TCM on dry eye, and found that there were some problems in the selection of TCM indicators of dry eye, such as low frequency of TCM indicators, large difference in index selection, nonstandard expression, poor clinical practicability, lack of long-term prognosis indicators and economic indicators. To obtain the index source of dry eye TCM core outcome sets, lay the foundation for the next step of constructing dry eye core outcome sets, so as to further standardize the clinical research of dry eye therapy with TCM, improve the recognition of the research results, and thus give full play to the advantages of TCM in the treatment of dry eye diseases.

## Author contributions

**Data curation:** Kang Tong, Qiao Ying Lian, Xing Yu Chen, Fang Chen.

**Formal analysis:** Kang Tong, Xing Yu Chen, Fang Chen.

**Funding acquisition:** Chaoyang Yang.

**Investigation:** Qiao Ying Lian, Wen Tang, Xing Yu Chen, Zhibin Wang, Shijie Qiao, Hairui Han.

**Resources:** Chaoyang Yang.

**Supervision:** Chaoyang Yang.

**Writing – original draft:** Kang Tong.

**Writing – review & editing:** Chaoyang Yang.
